# Supplementary material for: Emergence of metastability in frustrated oscillatory networks: the key role of hierarchical modularity
Source: Front Netw Physiol. 2024 Aug 21;4:1436046. doi: 10.3389/fnetp.2024.1436046 (PMC11372895; doi:10.3389/fnetp.2024.1436046)
Supplement: Supplementary file 1 [file DataSheet1.pdf]

# Supplementary Material: Emergence of metastability in frustrated oscillatory networks: the key role of hierarchical modularity

Enrico Caprioglio and Luc Berthouze
Department of Informatics, University of Sussex, BN1 9RH Brighton, UK
{E.Caprioglio, L.Berthouze}@sussex.ac.uk

August 15, 2024

## 1 Supplementary Appendix SA: Hierarchical Network Model Derivation

We modeled the probability of connection in the upper layers as $p_2(H) = (\frac{1}{2} + \frac{H}{2})\gamma$ and $p_3(H) = (\frac{1}{2} - \frac{H}{2})\gamma$, where the factor $\gamma$ in both expressions is used to control the average degree of the system. Then, for $H = 0$ we have $p_2(0) = p_3(0) = \gamma/2$ and for $H = 1$ we obtain $p_2(1) = \gamma$ and $p_3(1) = 0$. Given these constraints and using $n_3 = 2$ as in our model, we may write the average degree of each node as:

$$\begin{aligned}
\langle k \rangle &= (n_1 - 1)p_1(H) + (n_2 - 1)n_1 p_2(H) + (n_3 - 1)n_1 n_2 p_3(H) \\
&= (n_1 - 1)p_1(H) + (n_2 - 1)n_1 p_2(H) + n_1 n_2 p_3(H) \\
&= (n_1 - 1)p_1(H) + (n_2 - 1)n_1 \left[\left(\frac{1+H}{2}\right)\gamma\right] + n_1 n_2 \left[\left(\frac{1-H}{2}\right)\gamma\right] \\
&= (n_1 - 1)p_1(H) + n_1 n_2 \gamma - \frac{H+1}{2} n_1 \gamma,
\end{aligned}$$

where in the first step we used $n_3 = 2$ which we used in this study. By requiring the derivative of $\langle k \rangle$ with respect to $H$ to be zero we obtain

$$\begin{aligned}
\frac{\mathrm{d}\langle k \rangle}{\mathrm{d}H} &= (n_1 - 1)\frac{\mathrm{d}p_1}{\mathrm{d}H} - \frac{n_1}{2}\gamma = 0 \\
\frac{\mathrm{d}p_1}{\mathrm{d}H} &= \frac{n_1}{2(n_1 - 1)}\gamma \\
\Rightarrow p_1 &= \frac{n_1}{2(n_1 - 1)}\gamma H + C.
\end{aligned}$$

Note that $C$ is arbitrary. However, since $p_1 \in [0, 1]$, $H \in [0, 1]$, and we required $p_1$ to be as close to unity as possible, we may define $C$ to be equal to

$$C = 1 - \frac{n_1}{2(n_1 - 1)}\gamma,$$

without loss of generality. Finally, we rewrite $p_1$ as

$$p_1(H) = 1 - \left(\frac{n_1 \gamma}{n_1 - 1}\right)\left(\frac{1-H}{2}\right).$$

# 2 Supplementary Figures

## 2.1 Sensitivity Analysis

The regions of the parameter space in which chimera states are detected is found by computing the average and standard deviation of the difference between the populations' local KOPs across a simulation. We report the values we found as a function of $H$ averaged over 100 realizations for fixed $k = 51.2$ in the figures below, where we show how the regions limits vary depending on the choice of the arbitrary threshold while leaving our results qualitatively invariant.

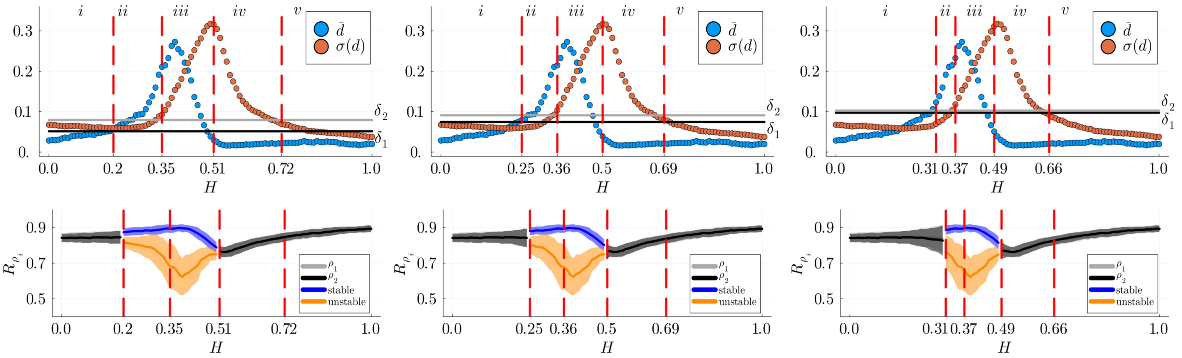


Figure SA10: Top row: Average and standard deviation of the difference between the populations' local KOPs, $\bar{d}$ and $\sigma(d)$ respectively, as a function of $H$ for fixed $k = 51.2$. The threshold values $\delta_1$, $\delta_2$ were chosen to be $s$ standard deviations higher than the baseline values obtained for $H = 0.0$. Left, middle and right panels show the regions identified when using $s = 1$, $s = 2$ and $s = 3$ respectively. Whilst the boundaries change, the results in Sec. 3.2 do not qualitatively change (bottom row).

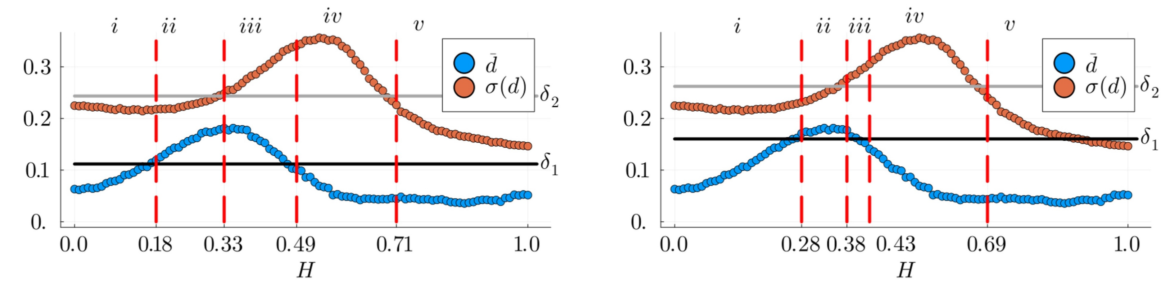


Figure SA11: Average and standard deviation of the difference between the populations' local KOPs, $\bar{d}$ and $\sigma(d)$ respectively, as a function of $H$ for fixed $k = 21$. The threshold values $\delta_1$, $\delta_2$ were chosen to be $s$ standard deviations higher than the baseline values obtained for $H = 0.0$. Left and right columns show the regions identified for $s = 1$ and $s = 2$, respectively. For higher values of $s$ stable and breathing chimeras are not detected.

## 2.2 Alternating Chimera States

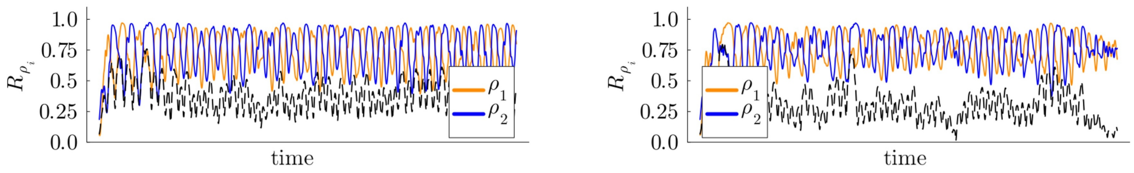


Figure SA12: Alternating chimera can be found for values of $H > 0.5$, for instance $H = 0.55$ (left) and $H = 0.58$ (right).

## 2.3 Metastability as a function of the average degree

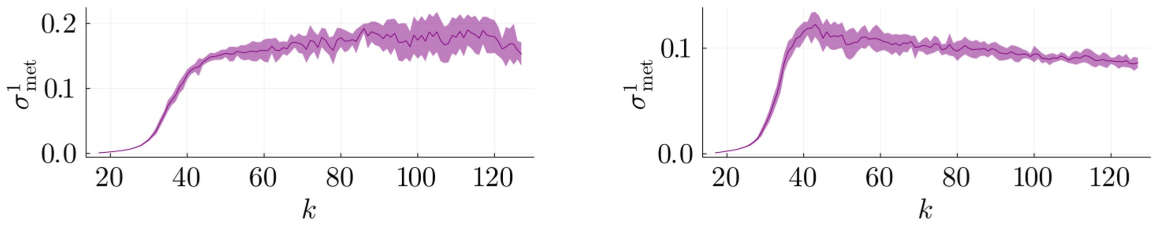


Figure SA13: Metastability in layer 1, $\sigma^1_{\mathrm{met}}$, as a function of $k$ for fixed $H = 0.5$ (left) and $H = 0.3$ (right).

## 2.4 Configuration model with phase-lags

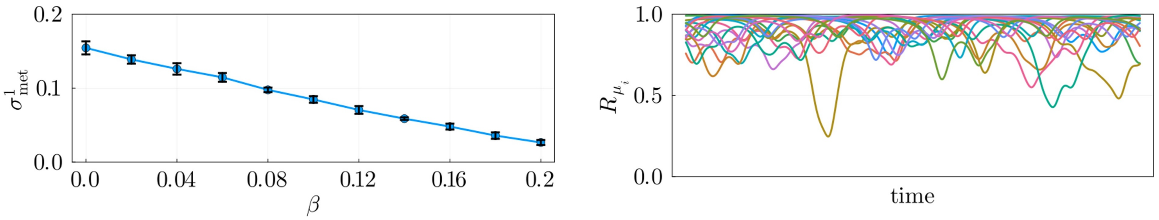


Figure SA14: Left: Metastability as a function of the lag parameter in random networks of oscillators constructed using the configuration model with average degree $k = 51$. Only pairs of oscillators which are associated with a different subset $c$ as defined by the partition vector $P_1$ share a phase-lagged interaction with phase-lag $\alpha = \pi/2 - \beta$. Right: example of modules' local KOP dynamics in time. Simulations performed using Euler method with step size $10^{-3}$ for 25000 steps. Metastability values and error bars obtained from 10 random initialization.

## 2.5 All phase-lagged interactions

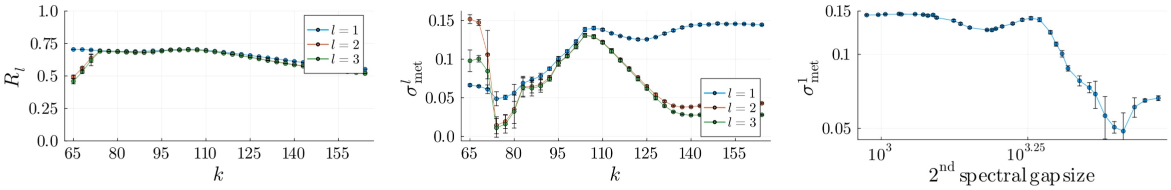


Figure SA15: Left: Average degree of synchronization $R_l$ at each layer $l$ as a function of the average degree $k$. Middle: Metastability indexes $\sigma^l_{\mathrm{met}}$ at each layer $l$ as a function of the average degree $k$. Right: Index of metastability in layer 1 for increasing second spectral gap size. Systems constructed using the variation of the nSBM with $n_1 = 64$. Values and error bars obtained from 10 random initialization.
